# hypeR-GEM: connecting metabolite signatures to enzyme-coding genes via genome-scale metabolic models

**DOI:** 10.1093/bioinformatics/btag588

**Published:** 2026-08-08

**Authors:** Ziwei Huang, Thomas Perls, Paola Sebastiani, Daniel Segrè, Stefano Monti

**Affiliations:** Department of Physics, Boston University, Boston, MA 02215, United States; Division of Computational Biomedicine, Boston University Chobanian & Avedisian School of Medicine, Boston, MA 02118, United States; Department of Medicine, Boston University Chobanian & Avedisian School of Medicine and Boston Medical Center, Boston, MA 02118, United States; Institute for Clinical Research and Health Policy Studies, Tufts Medical Center, Boston, MA 02111, United States; Department of Medicine, School of Medicine, Tufts University, Boston, MA 02111, United States; Data Intensive Study Center, Tufts University, Boston, MA 02111, United States; Department of Physics, Boston University, Boston, MA 02215, United States; Department of Biology, Boston University, Boston, MA 02215, United States; Department of Biomedical Engineering, Boston University, Boston, MA 02215, United States; Bioinformatics Program, Faculty of Computing and Data Science, Boston University, Boston, MA 02215, United States; Biological Design Center, Boston University, Boston, MA 02215, United States; Division of Computational Biomedicine, Boston University Chobanian & Avedisian School of Medicine, Boston, MA 02118, United States; Bioinformatics Program, Faculty of Computing and Data Science, Boston University, Boston, MA 02215, United States; Department of Biostatistics, Boston University School of Public Health, Boston, MA 02118, United States

## Abstract

**Motivation:**

Enrichment analysis is a cornerstone of “omics” data interpretation, enabling researchers to connect analysis results to biological processes and generate testable hypotheses. Enrichment analysis in metabolomics poses distinct challenges for interpretation and multi-omics integration due to the lack of well-defined and consistent connections to well-curated gene-centered biological knowledge repositories. To address these challenges, we developed *hypeR-GEM*, a methodology and associated R package that adapts gene set enrichment analysis to metabolomics. *hypeR-GEM* leverages genome-scale metabolic models (GEMs) to infer reaction-based links between metabolites and enzyme-coding genes, enabling the mapping of metabolite signatures to gene signatures and their subsequent annotation via gene set enrichment analysis.

**Results:**

We validated *hypeR-GEM* using paired metabolomics–proteomics and metabolomics–transcriptomics datasets by assessing whether genes mapped from metabolites significantly overlapped with differentially expressed proteins or transcripts. We further evaluated whether pathways enriched via *hypeR-GEM*-mapped genes corresponded to those derived from paired proteomic or transcriptomic data. In most datasets analyzed, both the predicted enzyme-coding genes and the associated enriched pathways showed significant concordance with independently derived omics signatures, supporting the utility and robustness of *hypeR-GEM*. Finally, we applied *hypeR-GEM* to the analysis of age-associated metabolic signatures from the New England Centenarian Study. The results revealed consistent enrichment of lipid-related pathways, aligning with the well-established role of lipid metabolism in aging, and highlighted additional pathways not captured in the metabolites’ annotation, demonstrating *hypeR-GEM*’s practical utility in a real-world use case.

**Availability and implementation:**

The *hypeR-GEM* R package, documentation, and workflow examples are freely available at https://github.com/montilab/hypeR-GEM and archived at https://doi.org/10.5281/zenodo.20586748.

## 1 Introduction

Metabolomics is the comprehensive study of metabolites in cells, tissues, or organisms, integrating advanced analytical technologies with statistical and computational tools to identify and quantify these molecules and extract biologically meaningful information (Idle and Gonzalez 2007, Roessner and Bowne 2009). A critical step in interpreting high-dimensional omics data is enrichment analysis, which reduces data complexity and enhances interpretability by identifying biologically relevant processes (Khatri *et al.* 2012, Marco-Ramell *et al.* 2018). Several metabolomic-specific platforms, such as MPEA (Kankainen *et al.* 2011) and MBRole (Chagoyen and Pazos 2011), provide metabolite-based enrichment analysis but lack capabilities for multi-omics integration. Statistical frameworks like Metabox (Wanichthanarak *et al.* 2017), Metascape (Karnovsky *et al.* 2012), and 3Omics (Kuo *et al.* 2013) address the integration of transcriptomic, proteomic, metabolomic data within metabolic pathway contexts and offer joint visualization functionalities; however, the enrichment analyses remain metabolite-centric and are restricted to a limited set of predefined metabolite pathways. MetExplore (Cottret *et al.* 2018) facilitates the multi-omics integration by leveraging genome-scale metabolic models (GEMs) to design an automated workflow for the customization and curation of GEMs based on user-defined metabolite inputs. However, it does not support metabolite-to-gene mapping and gene-based enrichment analysis. MetaboAnalyst (Xia *et al.* 2009, Pang *et al.* 2022) supports enrichment analyses across multiple omics layers, but only when users also provide transcriptomic or proteomic data, and each layer is analyzed independently. Although it provides joint visualization when multi-omics are available, it cannot infer gene- or protein-level information when only metabolomics data are provided. Overall, most of the existing metabolomics-focused frameworks perform enrichment analyses directly in the metabolite space. While this approach has useful applications, it does not support the connection to the extensive gene-centered biological knowledge and functional annotations available for transcriptomics and proteomics, which would enable a more comprehensive multi-omics integration.

Gene set enrichment based on over-representation analysis (ORA) is a widely used framework for interpreting transcriptomics data, supported by a large suite of analytical tools and curated molecular signature databases derived from existing biological knowledge (Subramanian *et al.* 2005, Irizarry *et al.* 2009, Hung *et al.* 2012). This framework has also been successfully extended to the analysis of proteomics data (Schmidt *et al.* 2014). However, applying gene-based ORA to metabolomics remains challenging due to the lack of well-defined and consistent associations between metabolites and genes (Schmidt 2004, Booth *et al.* 2013, Cavill *et al.* 2016).

To bridge this gap, we developed *hypeR-GEM*, an R package and methodology that harnesses the rich connectivity encoded in GEMs. GEMs are curated resources that capture all known metabolic components and interactions within a biological system, including metabolites, genes, enzymes, and reactions (Duarte *et al.* 2007, Sigurdsson *et al.* 2010, Brunk *et al.* 2018, Robinson *et al.* 2020). By leveraging the gene–reaction–metabolite associations within GEMs, *hypeR-GEM* establishes reaction-based mapping between metabolites and enzyme-coding genes, enabling ORA to be applied to metabolomics data through predicted gene-level annotations. Notably, GEMs encode comprehensive metabolic information that captures the complex interdependencies among genes, proteins, and metabolites, providing a multidirectional platform for connecting different omics layers. For example, Richelle *et al.* (2021) demonstrated the utility of GEMs in the opposite direction, inferring metabolic task activity directly from transcriptomic data. Similarly, Zheng *et al.* (2025) used GEMs to uncover biologically meaningful metabolite-mediated cell–cell communication from single-cell RNA-seq data, while Wagner *et al.* (2021) leveraged GEMs to characterize cellular metabolic states at single-cell resolution. These studies highlight the versatility of GEMs for integrating and interpreting multi-omics data across diverse biological contexts.

We evaluated the performance of *hypeR-GEM* using paired metabolomics–proteomics and metabolomics–transcriptomics datasets. Specifically, we tested whether enzyme-coding genes mapped from differential metabolomics signatures significantly overlapped with differentially expressed (DE) proteins or genes in the corresponding proteomics/transcriptomics datasets. We further assessed whether pathways enriched via ORA using *hypeR-GEM*-mapped genes aligned with pathways enriched in the signatures derived from the proteomic or transcriptomic data.

Our results demonstrate that, across multiple datasets, both the predicted genes and their enriched pathways show significant overlap with direct proteomic/transcriptomic signatures and their pathway enrichments. These findings validate *hypeR-GEM* as an effective tool for extending gene-centric enrichment analysis to datasets where only the metabolomics layer is available.

We conclude with a case study where we show the results of applying *hypeR-GEM* to age-associated metabolomics signatures from the New England Centenarian Study (NECS). This analysis revealed connections between enriched lipid-related pathways identified in metabolite-space, such as lipid and fatty acid metabolism, and those identified in gene-space via *hypeR-GEM*-mapped genes, including retinol, tryptophan, and bile acid metabolism. These findings illustrate *hypeR-GEM*’s ability to uncover previously validated biological mechanisms associated with aging.

## 2 Materials and methods

### 2.1 Algorithm framework

**Figure 1:**
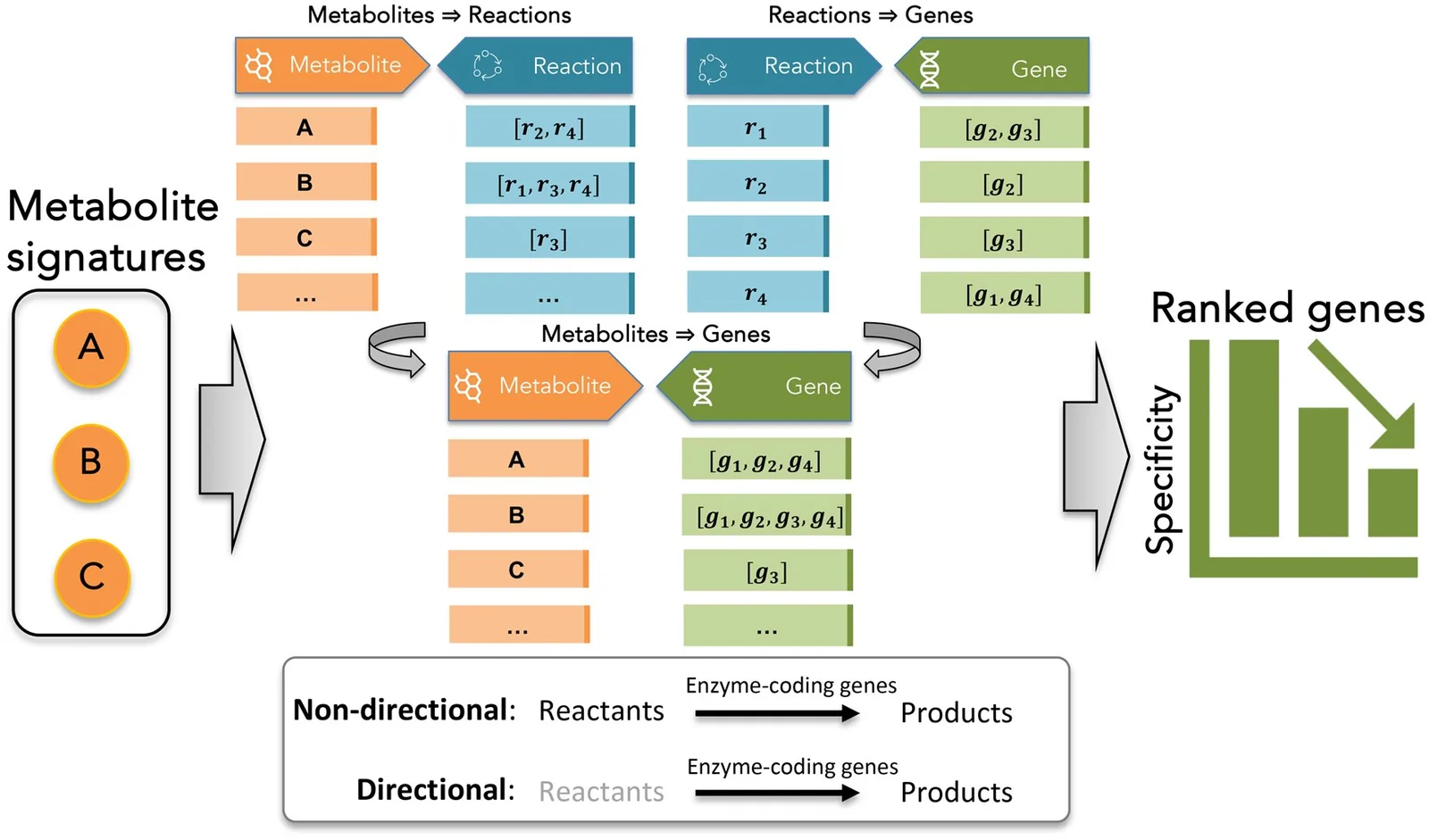
Overview of the *hypeR-GEM* algorithm. In the schematic, metabolites are denoted by capital letters (e.g. A, B), metabolic reactions by lowercase r with subscripts (e.g. $r_{1}$, $r_{2}$), and enzyme-coding genes by lowercase with subscripts (e.g. $g_{1}$, $g_{2}$). Given an input metabolite signature, *hypeR-GEM* identifies all reactions in which each metabolite participates and the corresponding enzyme-coding genes that catalyze these reactions. Two mapping strategies are supported: (i) *non-directional* mapping, linking metabolites through all associated reactions, and (ii) *directional* mapping, restricting associations to reactions in which the metabolite acts as a product. Each mapped gene is subsequently evaluated based on a gene-specificity hypergeometric test to assess the significance of its association with the input signature. The algorithm outputs a ranked list of enzyme-coding genes ordered by their association specificity.

The core of *hypeR-GEM* is a reaction-centric mapping algorithm that systematically links metabolites to enzyme-coding genes based on the biochemical relationships encoded within GEMs (Fig. 1). Given an input metabolic signature, a set of metabolites annotated using the RefMet nomenclature, *hypeR-GEM* identifies all metabolic reactions in which each metabolite participates as either reactant or product.

For each reaction (excluding exchange reactions and a subset of transport reactions), the associated enzyme-coding genes are then mapped to the metabolites according to two configurable rules: a *non-directional* mapping that considers all reactions involving the metabolite, and a *directional* mapping that restricts associations to reactions producing the metabolite based on the stoichiometry encoded in the model.

*Non-directional* mapping provides a comprehensive representation of metabolite–gene associations and is suited for exploratory analyses. In contrast, *directional* mapping restricts associations to reactions in which the metabolite is produced, enabling a more mechanistic interpretation focused on the upstream enzymatic processes responsible for metabolite production.

In addition, to mitigate the impact of highly connected metabolites (e.g. currency metabolites), *hypeR-GEM* allows optional filtering based on a user-defined promiscuity threshold (i.e. number of associated genes per metabolite), as implemented in the *signature2gene()* function. For reversible reactions, metabolites may be treated as both reactants and products; however, gene–metabolite associations are recorded as unique pairs, and no duplication or double-counting of genes is introduced.

To account for the lack of compartmental resolution in metabolomics data, compartment-specific (e.g. cytosolic versus mitochondrial) metabolites in GEMs can be optionally merged into a unified representation via the “merge” parameter in the *signature2gene()* function. This also prevents over-counting metabolite–gene associations across compartments, which would otherwise bias the *gene-specificity scores*.

To quantify the specificity of each mapped gene’s association to the input signature, *hypeR-GEM* computes a *gene-specificity score* based on the hypergeometric distribution. This test statistically assesses the overlap between signature metabolites and those linked to each gene via GEM reactions. The resulting *gene-specificity scores* are used as weights in a hypergeometric test for gene set overrepresentation analysis, thereby prioritizing genes with highly specific connections to the input metabolite signature.

### 2.2 Human-GEM

In this study, we utilized the open-source human GEM (Human-GEM V1.17.0) for analysis and validation. Human-GEM encompasses 13 026 metabolic reactions and 8365 metabolites across various compartments (e.g. cytosol, extracellular, etc.) and includes 3068 enzyme-coding genes. The model is accessible at https://github.com/SysBioChalmers/Human-GEM. Notably, we used the model solely as a curated knowledge base to obtain gene–reaction–metabolite associations and to map metabolites to enzyme-coding genes.

In addition to the human model, the information of GEMs for five major model organisms—Mouse, Rat, Zebrafish, Fruitfly, and Worm—was also extracted and integrated into *hypeR-GEM*. Further details regarding these models can be found in Wang *et al.* (2021b).

### 2.3 Reaction-based metabolite-to-gene mapping

A prototypical reaction in GEMs comprises three fundamental components: reactants, representing a set of metabolites consumed by the reaction; products, comprising a set of metabolites produced by the reaction; and enzymes, which are responsible for catalyzing the reaction (Henry *et al.* 2010, Gu *et al.* 2019). This gene–reaction–metabolite relationship provides a natural connection between metabolites and enzyme-coding genes. Specifically, for each metabolite of interest, we identified the reactions in which the metabolite participates, determined its roles (as reactant or product), and identified the genes encoding the corresponding enzymes catalyzing these reactions. Because *hypeR-GEM* uses GEMs solely as a curated repository of gene–reaction–metabolite relationships rather than for predicting reaction activity, we do not explicitly evaluate the Boolean gene–protein–reaction (GPR) rules that distinguish isoenzymes (“OR”) from multi-subunit enzyme complexes (“AND”) at the mapping stage. Instead, all genes listed in the GPR annotation for a reaction are included as associated enzyme-coding genes, reflecting metabolic association rather than context-specific catalytic activity. This design ensures that all genes metabolically linked to a metabolite through GEM reactions are considered, without imposing additional assumptions about reaction activity in the absence of context-specific omics data (e.g. transcriptomics or proteomics). We then established two mapping rules: a *non-directional* rule, mapping the metabolite to the enzyme-coding genes in a reaction if the metabolite serves as either a reactant or a product of that reaction; and a *directional* rule, mapping the metabolite to the genes only if the metabolite acts as a product of the reaction. For reversible reactions, metabolites may be treated as both reactants and products; however, gene–metabolite associations are recorded as unique pairs, and no duplication or double-counting of genes is introduced. By iteratively applying these mapping rules to each metabolite within the signatures, the signatures can be transformed from metabolite-space to gene-space.

### 2.4 Gene specificity

**Figure 2:**
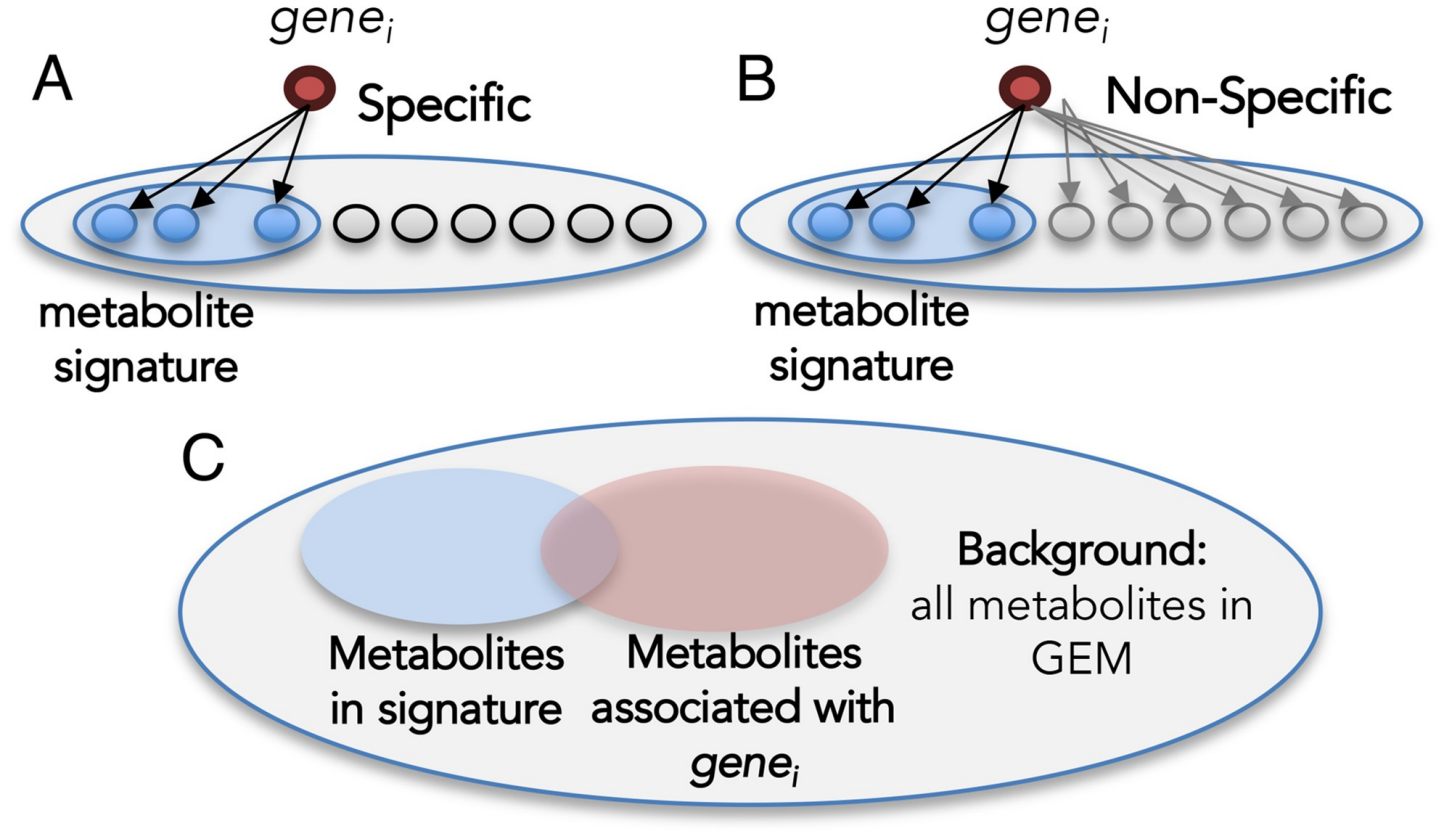
Illustration of gene-specificity. (A) Enzyme-coding genes that catalyze a limited number of reactions exhibit higher specificity in their association with metabolic signatures. (B) In contrast, non-specific enzymes, which catalyze more reactions, lead to more promiscuous gene–reaction–metabolite connections. (C) gene-specificity hypergeometric test quantifies the specificity of a given gene by assessing the statistical significance of the overlap between metabolites associated with the gene and those within a specific metabolic signature. The background for this test is defined as the total number of metabolites in the Human-GEM.

Enzyme-coding genes are present in all living cells, where they catalyze and regulate reactions essential to the existence of the living system (Borriss 1987, Copeland 2023). One important feature of enzymes is called reaction specificity, which depends on the shape of their active site (Cornish-Bowden 1984, Toney 2005, Bhatia and Bhatia 2018). Non-specific enzymes can catalyze a wide range of reactions, resulting in promiscuous gene–reaction–metabolite connections, and consequently, less specific associations with a given metabolic signature. To address this, we compute a *gene-specificity score* based on the hypergeometric distribution to evaluate the association specificity of each enzyme-coding gene (see Fig. 2). The computed score measures the statistical significance of the overlap between the metabolites associated with the gene across the entire GEM and those within the metabolite signature (Fig. 2).

For each gene $g_{i}$, let M denote the total number of metabolites represented in the GEM, S the number of metabolites in the metabolic signature, $A_{i}$ the number of metabolites associated with $g_{i}$ and $a_{i}\;$the number of those metabolites that overlap with the signature. The significance score $s_{i}$ is defined as the upper-tail hypergeometric probability of observing at least $a_{i}$ overlapping metabolites by chance:


(1) si=P(X ≥ ai)= ∑x=aimin⁡(Ai,S)(Aix)(M-AiS-x)(MS)

Smaller values of $s_{i}$ indicate more specific association. To incorporate this score into downstream enrichment analysis, each gene is assigned a weight $w_{i}\in [0,\;1]$ derived from a sigmoid transformation of its significance score:


(2) $$w_{i}=\frac{1}{1+e^{\left(a(\log_{10}(s_{i})-b)\right)}}$$

The sigmoid function is monotonic and bounded within [0, 1], assigning larger weights to genes with higher specificity. The parameter b determines the inflection point of the curve (the *half-point*, where $w_{i}=0.5$), allowing users to control the weight threshold, while a>0 regulates the steepness of the curve.

### 2.5 Weighted hypergeometric test

The standard hypergeometric test employed in ORA assesses whether a set of genes exhibits an over-representation of genes belonging to a specific category (e.g. pathway or ontology term), assuming that each gene contributes equally to the enrichment (Hahne *et al.* 2008, Qureshi and Sacan 2013). However, as shown in the Section 2.4, GEM-mapped enzyme-coding genes vary in their association specificity with the input metabolic signature. To incorporate this information into enrichment analysis, we extend the standard test to a weighted hypergeometric test that uses the gene-specificity weights $w_{i}$.

Formally, the probability of finding genes in a particular gene set or pathway is given by:


(3) P(X≥k|n;N;k)= ∑j=kn(Kj)(N-Kn-j)(Nn)

In the conventional formulation, N represents the size of background, n is the number of GEM-mapped enzyme-coding genes, K is the number of genes in each gene set or pathway, and k is the size of overlap.

In the weighted formulation, we first compute the continuous, weight-adjusted counts:


(4) $$n_{w}=\sum_{i\in n}w_{i},k_{w}=\sum_{i\in k}w_{i}.$$

Because the hypergeometric test requires integer inputs, these continuous counts are rounded to the nearest integer using the standard notation


(5) $$n_{w}=\left\lfloor \frac{1}{2}+\sum_{i\in n}w_{i}\right\rfloor ,k_{w}=\left\lfloor \frac{1}{2}+\sum_{i\in k}w_{i}\right\rfloor ,$$

where $\left\lfloor .\right\rfloor$ denotes the floor operator.

By substituting $n_{w}$ and $k_{w}\;$into the hypergeometric test formula, the weighted version effectively down-weights highly promiscuous genes that exhibit low specificity, thereby mitigating noise, reducing spurious enrichments, and improving the biological relevance of the enrichment results.

### 2.6 Implementation

*HypeR-GEM* is implemented entirely in R (R Core Team 2025). The core function, *signature2gene()*, accepts one or more input metabolic signatures and maps them to enzyme-coding genes using reaction-level information from GEMs. Input metabolites must be annotated using the RefMet nomenclature (Fahy and Subramaniam 2020). The output is a structured list object containing metadata required for downstream gene set ORA analysis, including Ensembl IDs (Hubbard *et al.* 2002), associated metabolites, the number of catalyzed reactions, and hypergeometric test statistics quantifying the signature-specificity of each mapped gene.

*HypeR-GEM* provides a modular workflow that facilitates not only the mapping of metabolites to genes, but also downstream gene set ORA (function *enrichment()*), visualization (functions *enrichment_plot(), rctbles(), sankey_plot()*), and generation of interactive reports (function *hypGEM_to_excel()*).

In addition, the *enrichment()* function provides a parameter (“min_metabolite”) that specifies the minimum number of distinct input metabolites required to support the mapped genes contributing to a given pathway. This constraint reduces the influence of highly connected metabolites (e.g. currency metabolites), ensuring that enriched pathways are supported by multiple distinct metabolite signals rather than driven by a single highly connected metabolite.

Users may apply their preferred statistical analysis pipelines to metabolomics data to derive statistically significant metabolite signatures, which can then be analyzed and interpreted in the gene space using *hypeR-GEM*. Output can be easily exported to Excel-compatible format, enabling reproducible and shareable reports.

### 2.7 Dataset overview

To evaluate the performance of *hypeR-GEM*, we utilized paired metabolomics–proteomics and metabolomics–transcriptomics datasets from 10 independent studies, covering four specimen categories and conducting differential analyses across 11 phenotypes (see Table 1). Results from the differential analyses and GEM-mapping are provided in Data S1–S11, available as supplementary data at *Bioinformatics* online, with only molecules meeting the significance threshold (adjusted *P*-value < .05) reported.

**Table 1:** Overview of paired datasets used for method evaluation.^a^

Dataset	Pair	Metabolites	Genes/proteins	Phenotype	Specimen
EMT	Metabolomics + Proteomics	8325	6426	Epithelial vs. Mesenchymal	Cell
Serine Starvation	Metabolomics + Transcriptomics	318	15 393	Serine-restriction versus Control	Cell
NECS Age	Metabolomics + Proteomics	1052	4783	Age	Plasma
NECS EL	Metabolomics + Proteomics	1052	4783	EL versus not	Plasma
M005 Kidney	Metabolomics + Proteomics	759	5358	SnellDW versus SnellWT	Tissue
M005 Liver	Metabolomics + Proteomics	509	4019	SnellDW versus SnellWT	Tissue
M005 Gatroc	Metabolomics + Proteomics	947	903	SnellDW versus SnellWT	Tissue
M005 Plasma	Metabolomics + Proteomics	971	2384	SnellDW versus SnellWT	Plasma
ROSMAP	Metabolomics + Proteomics	1055	8817	NCI versus AD	Tissue
COVID Urine	Metabolomics + Proteomics	1033	4003	COVID-19 versus Control	Urine
COVID Serum	Metabolomics + Proteomics	903	1494	COVID-19 versus Control	Plasma

a *Pair*: The paired omics layers. *Metabolites*: The number of metabolites. *Genes/proteins*: The number of proteins. *Phenotype*: The phenotype used for differential analysis. *Specimen*: The biological specimen from which the samples were derived. *EL*: Extreme longevity. *SnellWT*: Snell wild type mice. *SnellDW*: Snell dwarf mice. *NCI*: No cognitive impairment. *AD*: Alzheimer dementia.

#### 2.7.1 TGFβ-induced epithelial-to-mesenchymal transition in MCF10A cells

Epithelial-to-mesenchymal transition (EMT) is a biological process in which polarized epithelial (E) cells lose their specialized features, pass through intermediate hybrid states (E/M), and acquire mesenchymal (M) characteristics. This transition regulates cell plasticity in contexts such as embryonic development, wound healing, fibrosis, and cancer (Nieto *et al.* 2016, Paul *et al.* 2023). For this study, we analyzed paired metabolomics and proteomics datasets obtained from MCF10A cells exposed to TGFβ across 10-time points, each with three biological replicates. Following Paul *et al.* (2023), cells from the first three time points were classified as epithelial (E), those from the last four time points as mesenchymal (M), and the remaining time points as intermediate (E/M). The metabolomics dataset comprised 8325 metabolites, while the proteomics dataset included 6426 proteins. Differential analyses were performed by comparing cells in the epithelial (E) state with those in the mesenchymal (M) state, using a mixed-effects model that considered biological replicates as a random effect (see Table 1). The raw data are publicly available through ProteomeXchange (Proteomics: accession PXD031071) and the National Metabolomics Data Repository (Metabolomics: PR001174), as described in Paul *et al.* (2023).

#### 2.7.2 Serine starvation in HSC3 cells

Oral squamous cell carcinoma is an aggressive malignancy with limited therapeutic options. Emerging evidence highlights the critical role of serine, a non-essential amino acid, in regulating cell fate decisions, and demonstrates that serine restriction can suppress tumor progression across several cancer models. In this study, we analyzed paired metabolomics and transcriptomics datasets comprising six samples obtained from HSC3 cells cultured under serine-restricted versus control conditions (three samples per condition). The metabolomics dataset comprised 318 metabolites, while the transcriptomics dataset comprised 15 393 transcripts (see Table 1). The RNA-seq data are available in the Gene Expression Omnibus (GEO; accession GSE286464), and the corresponding study is described in Jankowski *et al.* (2025).

#### 2.7.3 The NECS

The NECS provides a unique resource for the investigation of the biological factors contributing to extreme human longevity (EL) (Sebastiani *et al.* 2021). We leveraged previously generated datasets obtained from serum samples subjected to proteomics (Sebastiani *et al.* 2021) and untargeted metabolomics (Monti *et al.* 2026) profiling. The metabolomics dataset (Monti *et al.* 2025, 2026) included 1052 metabolites that passed quality control across 213 subjects, comprising 70 centenarians, 80 offspring, and 63 controls. The proteomics dataset (Sebastiani *et al.* 2021) included 4783 proteins across 224 subjects comprising 77 centenarians, 82 offspring, and 65 controls, with 205 subjects in common between the two datasets. These were subsequently analyzed to identify age- and EL-associated molecular signatures (see Table 1).

#### 2.7.4 M005

The M005 dataset originates from the NIA Longevity Consortium’s lifespan-extension studies in genetically heterogeneous UM-HET3 mice (Watanabe *et al.* 2023). It comprises paired metabolomic and proteomic profiles from kidney, liver, gastrocnemius muscle (Gastroc), and plasma specimens, providing a systems-level resource for investigating molecular mechanisms of aging and longevity. Differential analyses were performed across all four specimen types by comparing 17 Snell dwarf (SnellDW) mice, which carry a loss-of-function mutation in the Pit1 gene leading to reduced body size, delayed sexual maturation, and extended lifespan (Watanabe *et al.* 2023), with 18 wild-type (SnellWT) controls. The numbers of metabolites and proteins in the metabolomics and proteomics datasets are summarized in Table 1.

#### 2.7.5 ROSMAP

The Religious Orders Study (ROS), initiated in 1994, and the Rush Memory and Aging Project (MAP), initiated in 1997, are longitudinal cohort studies collectively referred to as ROSMAP (Bennett *et al.* 2018, Pérez-González *et al.* 2024). These cohorts investigate risk factors for cognitive decline, Alzheimer’s disease, and related health outcomes. The metabolomics dataset (Pérez-González *et al.* 2024), derived from brain tissue, included 1055 metabolites that passed the quality control across 521 subjects, comprising 155 individuals with no cognitive impairment (NCI), 197 individuals with Alzheimer’s dementia (AD), and the remaining subjects having varying degrees or alternative causes of cognitive impairment. The proteomics dataset (Johnson *et al.* 2018, Ping *et al.* 2018, Johnson *et al.* 2020, Ping *et al.* 2020), also generated from brain tissue, included 8817 proteins across 400 subjects, comprising 168 individuals with NCI, 109 individuals with AD, and the remaining subjects having varying degrees or alternative causes of the cognitive impairment. We performed differential analyses comparing individuals with NCI to those with AD (see Table 1).

#### 2.7.6 COVID-19

This dataset derives from a clinical study of hospitalized COVID-19 patients in Taizhou, China, which systematically profiled the urinary and serum proteomes and metabolomes of 71 patients with COVID-19 together with 27 healthy controls (Bi *et al.* 2022). DE metabolites and proteins were identified by comparing COVID-19 disease with healthy individuals. We utilized the publicly available COVID-19-associated metabolite and protein signatures reported in this study (Bi *et al.* 2022) (see Table 1).

### 2.8 Differential analysis and single molecule-level evaluation

Differential analysis for each dataset (excluding COVID-19 datasets) was conducted using linear regression in R. For each paired dataset, differential analysis was performed independently on each omics layer to identify DE molecules, or signatures. For the COVID-19 datasets, we utilized publicly available signatures that had been previously identified (Bi *et al.* 2022). The *P*-values of each molecule were adjusted using the Benjamini–Hochberg method, statistical significance was assessed using an adjusted *P*-value threshold of < .05. The resulting signatures from each omics were further classified as up- or down-regulated based on the sign of the estimated coefficient. Up- and down-regulated metabolites were then used separately as inputs for *hypeR-GEM* to obtain mapped enzyme-coding genes. The evaluation was carried out by performing a hypergeometric test to assess the overlap between the union of *hypeR-GEM*-mapped enzyme-coding genes and the union of up- and down-regulated proteins/genes obtained from the corresponding proteomics/transcriptomics data.

### 2.9 Gene set enrichment analysis and pathway-level evaluation

Gene set enrichment analysis was conducted using the *hypeR* (Federico and Monti 2020) package in R on pathways from the Hallmark (Liberzon *et al.* 2015), Kyoto Encyclopedia of Genes and Genomes (KEGG) (Kanehisa *et al.* 2017), REACTOME (Fabregat *et al.* 2018), Gene Ontology Biological Process (GOBP) (Ashburner *et al.* 2000), and Gene Ontology Molecular Function (GOMF) (Ashburner *et al.* 2000). This analysis was conducted independently on the enzyme-coding genes predicted by *hypeR-GEM* and signatures derived from the corresponding proteomic/transcriptomic signatures, with a significant threshold of adjusted *P*-value < .05. The evaluation employs hypergeometric test to assess the overlap between the enriched pathways identified from *hypeR-GEM*-mapped genes and those obtained from proteomics/transcriptomics signatures.

### 2.10 Application to age-associated metabolic signatures in NECS

We analyzed age-associated metabolic signatures derived from the NECS metabolomics dataset (Monti *et al.* 2026) and performed Metabolite Set Enrichment Analysis using pathway annotations from Metabolon, Inc. (Evans *et al.* 2009, Kennedy *et al.* 2017) and RefMet classification system (Fahy and Subramaniam 2020), identifying metabolite-level enriched pathways based on an adjusted *P*-value threshold of ≤ .05. Additionally, we applied *hypeR-GEM* to map these age-associated metabolites to enzyme-coding genes, followed by Gene Set Enrichment Analysis using curated gene sets from Hallmark (Liberzon *et al.* 2015) and KEGG (Kanehisa *et al.* 2017), identifying enriched gene-level pathways (adjusted *P*-value < .05).

To integrate these complementary layers into a unified biological context, we constructed three-layer Sankey diagrams connecting DE metabolites, the enriched metabolite sets (MSets), and the enriched gene sets (GSets) derived from *hypeR-GEM*. Edge weights between the metabolite and Msets layers reflect the number of shared metabolites, while edges between the MSets and GSets layers reflect the overlap between *hypeR-GEM*–mapped genes and genes in each GSets. This framework enables the joint visualization of age-associated mechanisms across both metabolite and gene/protein spaces.

## 3 Results

### 3.1 Algorithm overview

The *hypeR-GEM* algorithm employs a reaction-centric strategy to establish systematic connections between metabolites and enzyme-coding genes by leveraging the metabolic relationships encoded in GEMs (see Fig. 1). Within a GEM, each metabolite is associated with one or more reactions, in which it participates as either a reactant or a product. Each reaction, in turn, is catalyzed by one or more enzyme-coding genes, with exceptions such as exchange reactions and certain transport reactions that are not catalyzed by enzymes. This metabolite–reaction–gene triad provides a natural framework for mapping metabolites to genes. Specifically, a metabolite is associated to an enzyme-coding gene if the gene encodes an enzyme that catalyzes one or more reactions in which the metabolite is involved. In this mapping step, all metabolites, including currency metabolites such as ATP, NADH, H₂O, and H⁺, are treated equivalently, provided they do not exceed a user-defined promiscuity threshold (i.e. number of associated genes), thereby allowing highly connected metabolites to be optionally excluded.

To accommodate different biological contexts, *hypeR-GEM* supports both *non-directional* and *directional* mapping rules, which respectively connect metabolites through all reactions in which they participate or only through reactions in which they are produced, according to the stoichiometry encoded in the model (Orth *et al.* 2010). *Non-directional* mapping provides a comprehensive view of metabolite–gene associations and is suited for exploratory analyses, whereas *directional* mapping restricts associations to reactions in which the metabolite is produced, enabling a more mechanistic interpretation (see Section 2 for full details).

Each mapped gene is subsequently evaluated using a gene-specificity hypergeometric test to quantify the significance of its association with the input signature (see Section 2 for full details). The final output is a ranked list of enzyme-coding genes ordered by their association specificity as shown in Fig. 1.

### 3.2 Evaluation

We evaluated the performance of *hypeR-GEM* using paired metabolomics–proteomics and metabolomics–transcriptomics datasets from 10 independent studies: (i) TGFβ-induced EMT in MCF10A cells (Paul *et al.* 2023); (ii) Serine starvation in HSC3 cells (Jankowski *et al.* 2025); (iii) The NECS (Sebastiani *et al.* 2021, Monti *et al*. 2026); (iv)–(vii) Mouse kidney, liver, gastrocnemius muscle, and plasma from the M005 dataset (Watanabe *et al.* 2023); (viii) The ROS and Memory and Aging Project Study (ROSMAP) (Bennett *et al.* 2018, Pérez-González *et al.* 2024), and (ix) and (x) Urine and serum samples from healthy and COVID-19 patients (Bi *et al.* 2022) (see Section 2 for full details).

**Figure 3:**
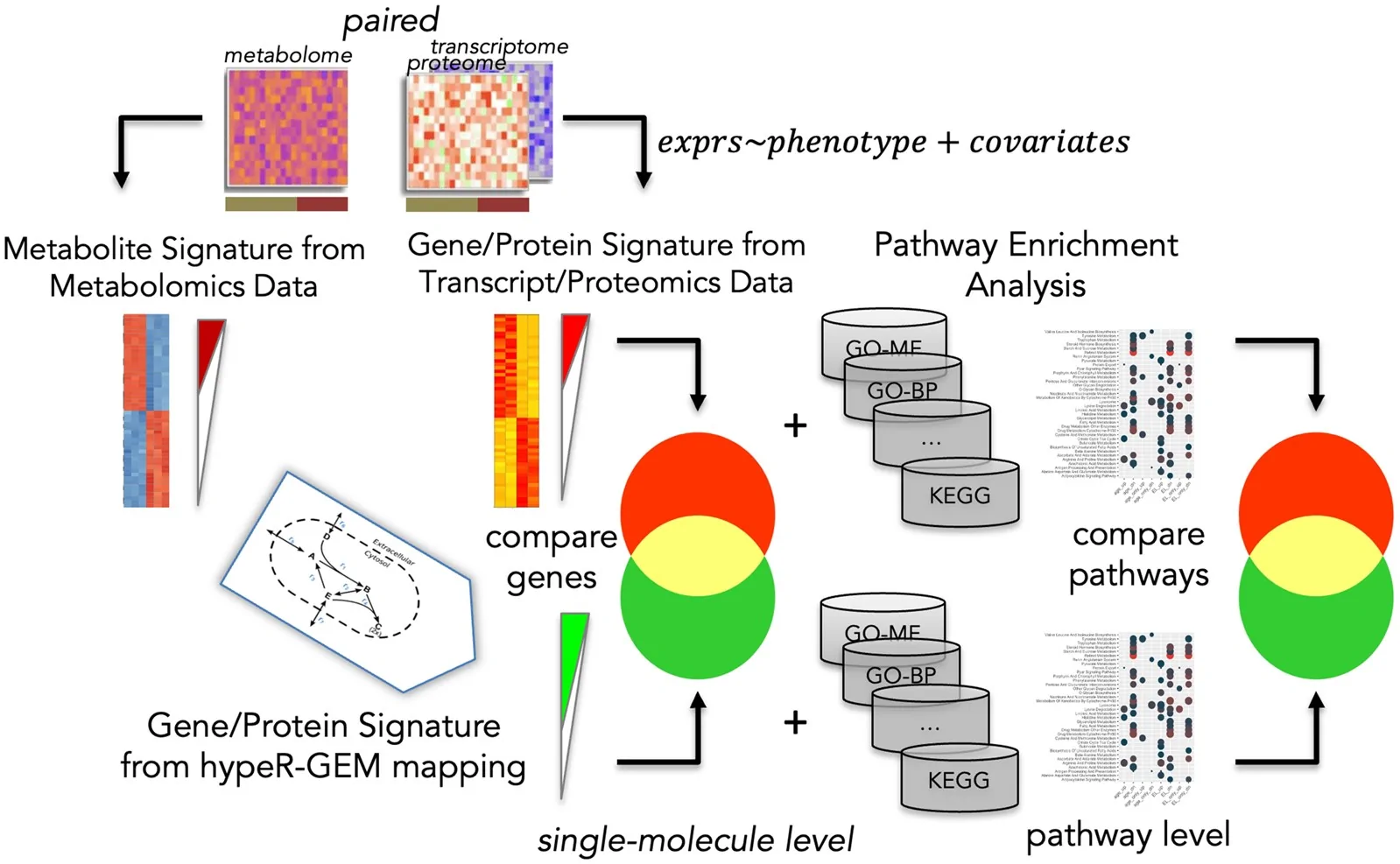
Schematic of method performance evaluation at single-molecule and pathway level. Differential analysis is independently performed on the paired metabolomics and transcriptomics or proteomics datasets to identify molecular signatures. Metabolite signatures are mapped to enzyme-coding genes via *hypeR-GEM*. At the single-molecule level, we evaluated the significance of the overlap between *hypeR-GEM*-mapped enzyme-coding genes (green/bottom set) and DE genes or proteins from the paired omics layer (red/top set). At the pathway level, *hypeR-GEM*-mapped genes and the corresponding transcriptomic/proteomic signatures are independently annotated by ORA analysis, and the significance of the overlap between their enriched pathways is assessed.

For each study, we conducted independent differential analyses on one or more phenotypes of interests using linear regression on both metabolomics and proteomics/transcriptomics data to identify molecular signatures (i.e. DE metabolites, proteins, or transcripts). In total, we analyzed 11 phenotypes across 10 datasets, covering four specimen categories (see Table 1). The metabolite signatures served as input for *hypeR-GEM*, which mapped them to enzyme-coding genes. Differential analysis and mapping results are provided in Data S1–S11, available as supplementary data at *Bioinformatics* online. The schematic flowchart of the method performance evaluation is shown in Fig. 3. We assessed performance at two levels:

*Single-molecule level*. We evaluated whether the mapped/predicted enzyme-coding genes significantly overlapped with the DE genes or proteins from the paired omics layer (see Section 2 for full details). Only comparisons where both omics layers yielded ≥ 10 significant molecules (with mapping in Human-GEM) were considered, as shown in Table 2.

**Table 2:** Evaluation of *hypeR-GEM*’s performance at single-molecule level.^a^

Dataset	DE metabolites	**hypeR-GEM** **(ND/D)**	DE genes	Overlap (ND/D)	*P*-value (ND/D)	Background
EMT	169	211/175	698	124/107	6.77e - 3/1.99e - 3	1376
Serine Starvation	36	672/577	833	273/233	2.14e - 3/7.63e - 3	2309
NECS Age	291	118/98	142	33/28	1.10e - 2/1.45e - 5	722
NECS EL	274	132/102	142	34/26	3.63e - 2/7.45e - 2	722
M005 Kidney	328	331/271	150	50/42	7.17e - 4/1.34e - 4	1482
M005 Liver	227	322/275	402	122/108	4.50e - 2/2.27e - 2	1291
M005 Gatroc	227	74/49	28	2/2	7.76e - 1/5.49e - 1	762
M005 Plasma	143	40/34	55	20/15	1.04e - 2/1.04e - 1	164
ROSMAP	61	169/128	152	23/11	2.99e - 2/6.46e - 1	1652
COVID Urine	367	163/125	94	28/26	4.89e - 3/3.25e - 5	859
COVID Serum	384	108/95	20	7/7	6.20e - 1/4.52e - 1	301

a *DE metabolites*: Number of DE metabolites in the metabolomics data. *hypeR-GEM (ND/D)*: Number of *hypeR-GEM* mapped enzyme-coding genes based on *non-directional* (ND) and *directional* (D) mapping. *DE genes*: Number of DE genes/proteins in the transcriptomics/proteomics data. *Overlap (ND/D)*: The overlap between “hypeR-GEM” and “DE Genes.” *P-value*: The *P*-value of the hypergeometric test. *Background*: The background parameter of the hypergeometric test, defined as the intersection between the proteins represented in GEM and those in the corresponding proteomic or transcriptomic dataset.

For each comparison, we report the number of DE metabolites, the number of enzyme-coding genes predicted by *hypeR-GEM* using both *non-directional* and *directional* mapping rules, and the number of DE genes/proteins from the paired omics layer. The *non-directional* and *directional* mapping strategies correspond to connecting metabolites through all reactions in which they participate or only through reactions in which they are produced, respectively (see Section 2). We further evaluated the significance of the overlap between the predicted enzyme-coding genes and DE genes/proteins via Fisher test, with the background defined as the intersection of enzyme-coding genes represented in Human-GEM and the genes/proteins measured in the paired omics layer.

**Figure 4:**
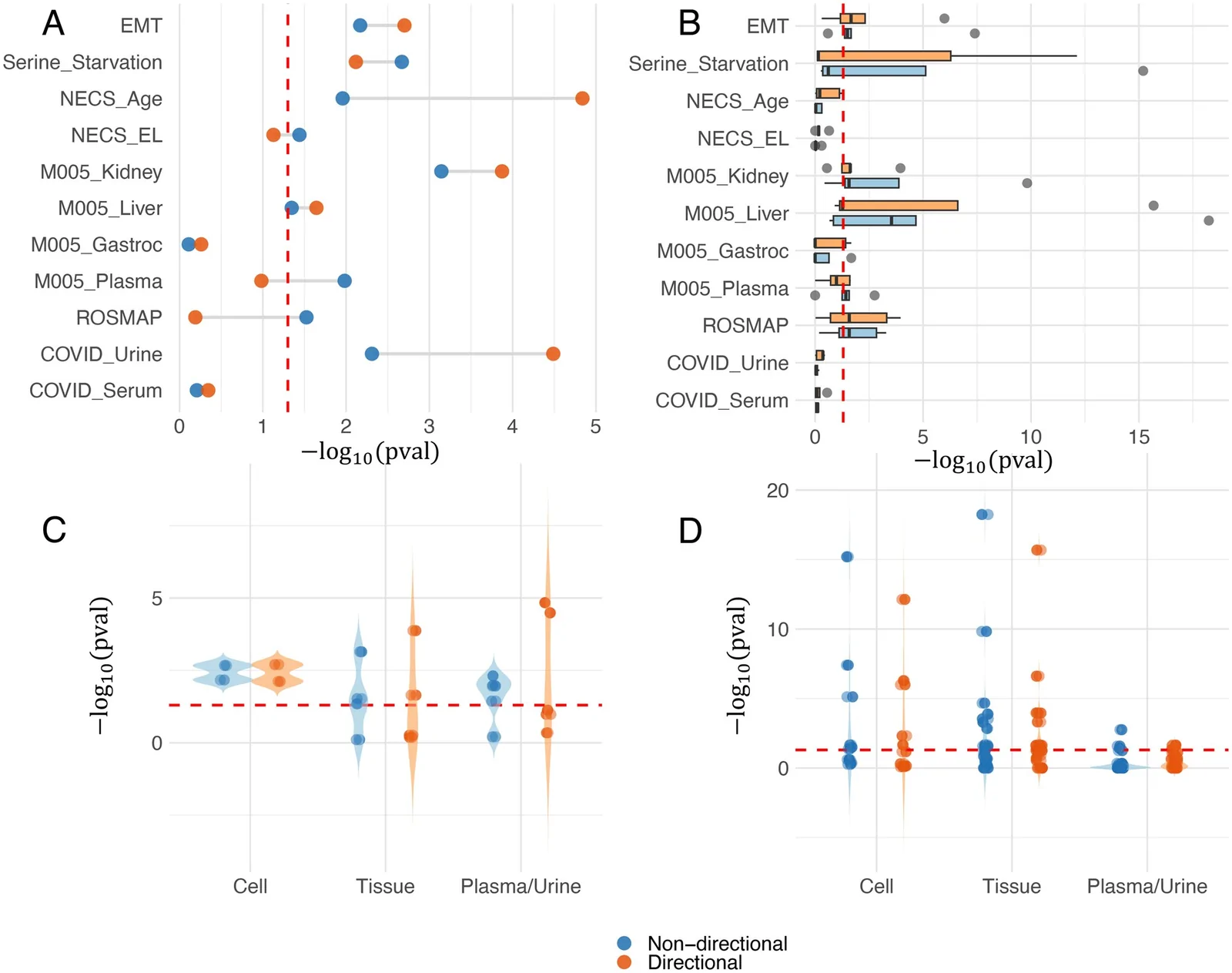
Single-molecule level and pathway level evaluation summary. The red dash line represents the α = 0.05 significance level. (A) Significance of the single-molecule level overlap between enzyme-coding genes predicted by *hypeR-GEM* and the corresponding proteomic or transcriptomic signatures for each dataset. (B) Significance of the pathway level overlap between enriched pathways identified by *hypeR-GEM* predicted enzyme-coding genes and those identified by annotation of the corresponding proteomic or transcriptomic signatures for each dataset. (C) Significance of the overlaps at the single-molecule level grouped by specimen type. (D) Significance of the overlaps at the pathway level grouped by specimen type.

A dataset-level summary is visualized in Fig. 4A, while Fig. 4C summarizes the results by specimen category. Notably, 9 out of the 11 comparisons exhibited statistically significant overlap (*P* < .05) under at least one mapping rule, supporting the effectiveness of *hypeR-GEM* at the single-molecule level. Significant overlaps were observed across all specimen categories. Strong concordance was particularly evident in cell-line and tissue datasets, including EMT, serine starvation, kidney, and liver, where the sources of molecular signals are relatively homogeneous. Importantly, comparisons involving plasma-based and urine samples also demonstrated statistically significant overlap, which further validate the practical utility of *hypeR-GEM*.

*Pathway level*. Gene set ORA analysis was independently performed on (i) *HypeR-GEM*-mapped enzyme-coding genes, and (ii) Corresponding proteomic or transcriptomic signatures. Pathway databases used included Hallmark (Liberzon *et al.* 2015), KEGG (Kanehisa *et al.* 2017), REACTOME (Fabregat *et al.* 2018), GOBP (Ashburner *et al.* 2000), and GOMF (Ashburner *et al.* 2000). We tested whether pathways enriched in signatures obtained from *hypeR-GEM*-mapped genes significantly overlapped with those obtained from the corresponding proteomic/transcriptomic data (see Section 2 for full details). Only comparisons where both omics layers yielded at least one enriched pathway were reported, see Table 3.

**Table 3:** Evaluation of *hypeR-GEM*’s performance at the pathway level.^a^

Dataset/geneset		hypeR-GEM (ND/D)	DE pathways	Overlap (ND/D)	% Overlap versus DE pathway (ND/D)	% Overlap versus hypeR-GEM (ND/D)	*P*-value (ND/D)	Background
EMT	HALLMARK	8/10	25	7/8	28/32	87.5/80	4.28e - 2/6.70e - 2	46
	KEGG	46/41	52	22/22	42.3/42.3	47.8/53.7	3.21e - 2/4.84e - 3	145
	REACTOME	40/40	68	15/15	22/22	37.5/37.5	2.16e - 2/2.16e - 2	292
	GOBP	404/372	762	151/136	19.8/17.8	37.3/36.6	3.97e - 8/1.02e - 6	2927
	GOMF	149/149	149	40/37	26.8/24.8	26.8/24.8	2.55e - 1/4.97e - 1	608
Serine Starvation	HALLMARK	9/10	34	8/7	23.5/20.6	88.9/70	2.45e - 1/7.71e - 1	46
	KEGG	55/51	44	17/14	38.6/31.8	30.9/27.5	4.43e - 1/7.08e - 1	150
	REACTOME	127/113	96	53/51	55.2/53.1	41.7/45.1	7.55e - 6/5.20e - 7	349
	GOBP	414/448	985	200/204	20.3/20.7	48.3/45.5	6.31e - 16/7.43e - 13	3206
	GOMF	166/163	147	32/28	21.8/19.0	19.3/17.2	5.41e - 1/8.11e - 1	762
NECS Age	HALLMARK	12/9	8	1/1	12.5/12.5	8.3/11.1	9.30e - 1/8.52e - 1	46
	KEGG	28/27	37	3/3	8.1/8.1	10.7/11.1	9.96e - 1/9.95e - 1	133
	REACTOME	24/25	29	4/7	13.8/24.1	16.7/28	4.60e - 1/4.52e - 2	202
	GOBP	362/312	634	71/71	11.2/11.2	19.6/27.5	5.71e - 1/3.87e - 1	2728
	GOMF	76/69	74	13/16	17.6/21.6	17.1/23.2	2.25e - 1/3.83e - 3	450
NECS EL	HALLMARK	13/13	8	1/2	12.5/25	7.7/15.4	9.47e - 1/7.34e - 1	46
	KEGG	33/28	37	4/3	10.8/8.1	12.1/10.7	9.96e - 1/9.96e - 1	133
	REACTOME	25/22	29	4/3	13.8/10.3	16/13.6	4.99e - 1/6.44e - 1	202
	GOBP	378/366	634	76/82	12.0/13.0	20.1/22.4	9.49e - 1/6.80e - 1	2728
	GOMF	93/75	74	11/15	14.9/20.2	11.8/20	9.38e - 1/2.26e - 1	450
M005 Kidney	HALLMARK	9/8	11	3/3	27.3/27.3	33.3/37.5	3.66e - 1/2.84e - 1	46
	KEGG	32/26	9	5/4	55.6/44.4	15.6/15.4	2.68e - 2/5.73e - 2	143
	REACTOME	32/26	8	3/3	37.5/37.5	9.4/11.5	4.16e - 2/2.36e - 2	303
	GOBP	333/288	264	64/44	24.2/16.7	19.2/15.3	1.50e - 10/1.11e - 4	2997
	GOMF	134/120	50	22/17	44/34	16.4/14.1	1.32e - 4/2.36e - 2	631
M005 Liver	HALLMARK	7/6	17	4/4	23.5/23.5	57.1/66.7	2.16e - 1/1.24e - 1	46
	KEGG	36/30	38	20/20	52.6/52.6	55.6/66.7	2.14e - 5/2.46e - 7	140
	REACTOME	29/24	27	5/5	18.5/18.5	17.2/20.8	1.42e - 1/7.26e - 2	273
	GOBP	296/241	251	74/62	29.5/24.7	25/25.7	5.75e - 19/2.08e - 16	2834
	GOMF	112/111	88	31/24	35.2/27.3	27.7/21.6	2.89e - 4/5.31e - 2	548
M005 Gastroc	HALLMARK	7/7	2	2/2	100/100	28.6/28.6	2.12e - 2/2.12e - 2	45
	KEGG	19/14	4	0/0	0/0	0/0	1/1	116
	REACTOME	15/13	2	0/0	0/0	0/0	1/1	169
	GOBP	202/165	60	7/5	11.7/8.3	3.5/3.0	2.28e - 1/3.87e - 2	2427
	GOMF	54/45	7	0/0	0/0	0/0	1/1	431
M005 Plasma	HALLMARK	6/4	3	2/2	66.7/66.7	33.3/50	5.59e - 2/2.47e - 2	38
	KEGG	7/6	4	3/2	75/50	42.9/33.3	3.76e - 2/1.91e - 1	28
	REACTOME	3/5	10	0/0	0/0	0/0	1/1	33
	GOBP	112/114	55	12/8	21.8/14.5	10.7/7.0	1.75e - 3/1.03e - 1	1295
	GOMF	35/33	4	3/3	75/75	8.6/9.1	2.65e - 2/2.23e - 2	173
ROSMAP	HALLMARK	11/10	17	4/2	23.5/11.8	36.4/20	6.51e - 1/9.53e - 1	46
	KEGG	32/29	34	11/9	32.3/26.5	34.4/31.0	7.73e - 2/1.94e - 1	146
	REACTOME	29/29	35	7/7	20/20	24.1/24.1	2.62e - 2/2.62e - 2	321
	GOBP	342/320	490	77/73	15.7/14.9	22.5/22.8	5.11e - 4/4.82e - 4	3062
	GOMF	103/90	110	28/28	25.5/25.5	27.2/31.1	1.44e - 3/1.09e - 4	678
Covid Urine	HALLMARK	4/4	8	0/0	0/0	0/0	1/1	46
	KEGG	31/37	12	1/4	8.3/33.3	3.2/10.8	9.64e - 1/4.33e - 1	134
	REACTOME	23/19	18	1/1	5.6/5.6	4.3/5.3	9.12e - 1/8.62e - 1	191
	GOBP	267/215	157	13/14	8.3/8.9	4.9/6.5	7.81e - 1/3.51e - 1	2732
	GOMF	73/74	29	4/5	13.8/17.2	5.5/6.8	6.37e - 1/4.35e - 1	499
Covid Serum	HALLMARK	4/6	2	0/0	0/0	0/0	1/1	44
	KEGG	22/17	4	1/1	25/25	4.5/5.9	7.21e - 1/6.13e - 1	82
	REACTOME	13/15	14	1/1	7.1/7.1	7.7/6.7	9.27e - 1/9.53e - 1	83
	GOBP	187/162	55	4/3	7.3/5.5	2.1/1.9	7.55e - 1/8.24e - 1	2050
	GOMF	49/53	5	1/2	20/40	2.0/3.8	6.59e - 1/2.78e - 1	255

a *hypeR-GEM (ND/D)*: Number of enriched pathways obtained from *hypeR-GEM* mapped enzyme-coding genes based on *non-directional* (ND) and *directional* (D) mapping. *DE pathways*: Number of enriched pathways obtained from transcriptomics/proteomics signatures. *Overlap (ND/D)*: The overlap between “hypeR-GEM” and “DE Pathways.” *% Overlap versus DE pathways (ND/D)*: Percentage of overlapping pathways relative to the number of DE Pathways. *% Overlap versus hypeR-GEM (ND/D)*: Percentage of overlapping pathways relative to the number of *hypeR-GEM* pathways. *P-value (ND/D)*: The *P*-value of the hypergeometric test. *Background*: The background parameter of the hypergeometric test, defined as the intersection of gene sets that are sufficiently represented in GEM and the corresponding proteomic or transcriptomic dataset.

For each comparison, we report the number of enriched pathways identified from *hypeR-GEM* predicted genes via both *non-directional* and *directional* mapping rules, as well as the number of enriched pathways derived from the DE genes/proteins in the paired omics layer. As in the single-molecule level evaluation, we applied a hypergeometric test to assess the statistical significance of the pathway-level overlap. The background for this test was defined as the set of pathways with sufficient representation in both Human-GEM and the corresponding transcriptomic or proteomic dataset.

A dataset-level summary is visualized in Fig. 4B, while specimen-level results are summarized in Fig. 4D. Notably, seven out of the nine comparisons that showed significant overlap at the single-molecule level also demonstrated statistically significant pathway-level overlap (*P* < .05) in at least one pathway database. Moreover, the M005 gastrocnemius muscle dataset, which did not show significant overlap at the single-molecule level, exhibited significant overlap in at least one pathway collection at the pathway level. In total, 8 out of 11 comparisons, spanning all specimen categories (cell, tissue, plasma, and urine), demonstrated statistically significant pathway-level concordance, further supporting the efficacy and generalizability of *hypeR-GEM* for functional interpretation of metabolomics data.

Global rank-based Kolmogorov-Smirnov tests were performed to assess whether the distributions of *P*-values were significantly skewed toward zero, with both the single-molecule and pathway level tests yielding highly significant results (Table 4).

**Table 4:** Global KS test.^a^

Level	Mapping	Statistics	*P*-value
Single-molecule	Non-directional	0.77	1.01E - 07
	Directional	0.62	5.90E - 05
Pathway	Non-directional	0.33	8.42E - 06
	Directional	0.34	2.32E - 06

a *Level*: Evaluation level. *Statistics*: The statistics of the KS test. *P*-value: The *P*-value of the KS test.

### 3.3 Application

**Figure 5:**
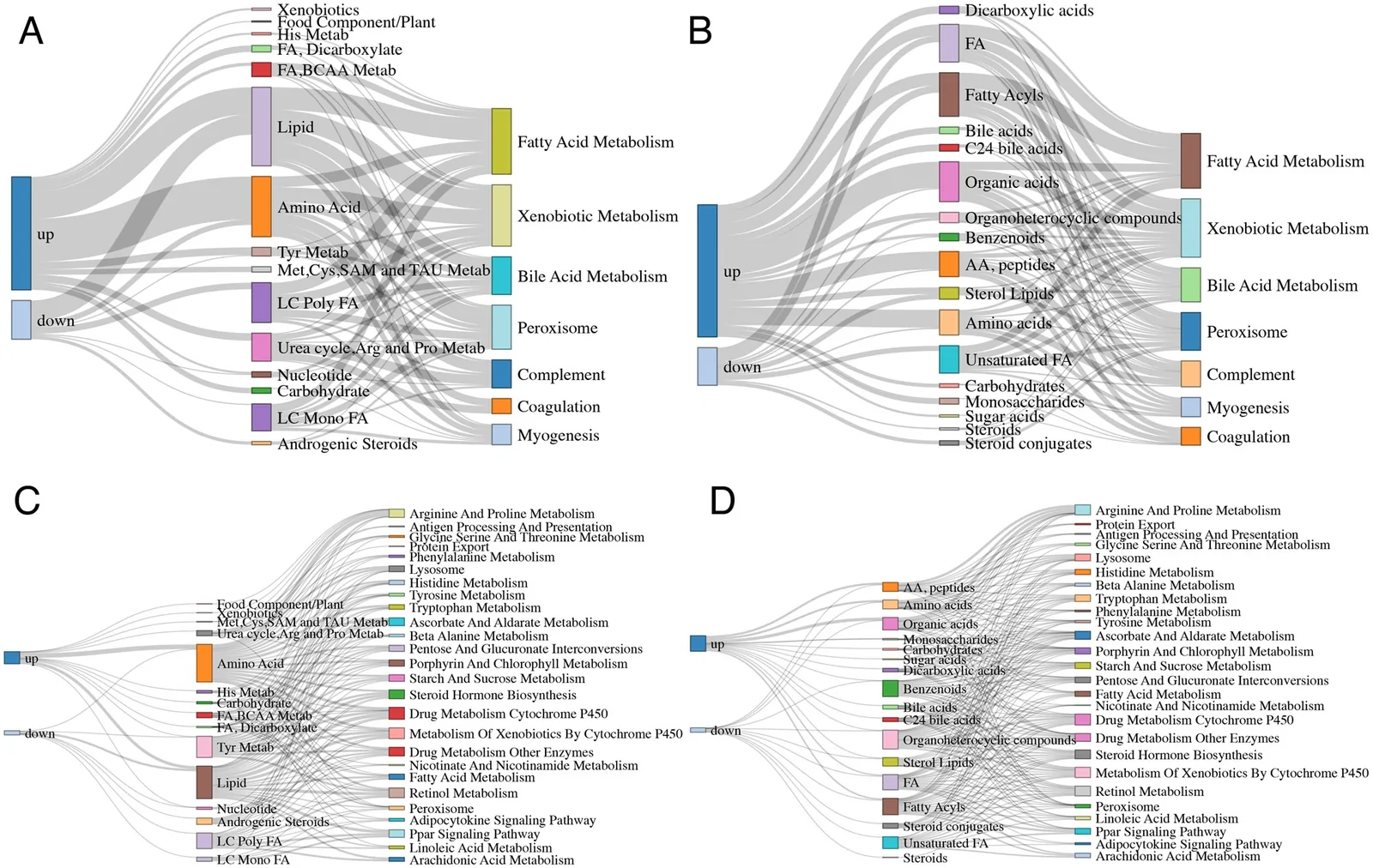
Metabolite- and gene-based annotation of age-associated signatures. Three-layer Sankey diagrams illustrating the connections between age-associated metabolites, enriched metabolite sets, and enriched gene sets (from left to right). Gene set ORA was performed using the HALLMARK and KEGG compendia. (A) Age-associated metabolites, Metabolon-annotated metabolite sets, and HALLMARK gene sets. (B) Age-associated metabolites, RefMet-annotated metabolite sets, and HALLMARK gene sets. (C) Age-associated metabolites, Metabolon-annotated metabolite sets, and KEGG gene sets. (D) Age-associated metabolites, RefMet-annotated metabolite sets, and KEGG gene sets.

We further demonstrate the practical utility of *hypeR-GEM* for multi-omics integration through a use-case using metabolomics data from the NECS (Monti *et al.* 2026). The dataset comprises 1213 measured and 888 annotated metabolites. A signature of age-associated metabolites was identified by regressing each metabolite on age while controlling for sex, batch and years of education. This analysis identified 209 up-regulated and 84 down-regulated metabolites (adjusted *P*-value < .05). Among the up-regulated metabolites, 84 were represented in GEM and mapped to 307 and 250 enzyme-coding genes under the *non-directional* and *directional* mapping rules, respectively. Similarly, of the 84 down-regulated metabolites, 29 were represented in GEM and mapped to 274 and 190 enzyme-coding genes under the two mapping schemes. ORA-based enrichment analyses were performed both in metabolite and gene space, and the results are summarized in the three-layer Sankey diagram shown in Fig. 5 (see Section 2 for full details), linking age-associated metabolites, enriched metabolite sets, and gene sets.

Age-associated metabolites were significantly enriched in multiple lipid-related pathways across both annotation systems—namely, “Lipid” and “Long Chain Polyunsaturated Fatty Acids (LC Poly FA),” “Long Chain Monounsaturated Fatty Acids (LC Mono FA),” “Fatty Acids, Dicarboxylate (FA, Dicarboxylate),” among others (Metabolon; Fig. 5A and C), as well as “Fatty Acids (FA),” “Unsaturated Fatty Acids (Unsaturated FA),” “Bile acids,” “Fatty Acyls,” and “Sterol Lipid,” among others. (RefMet; Fig. 5B and D). Notably, these metabolite-level lipid-based pathways are also linked to gene-level lipid-associated pathways enriched in *hypeR-GEM* predicted genes, including “Bile Acid Metabolism,” “Fatty Acid Metabolism,” “Retinol Metabolism,” “Steroid Hormones Biosynthesis,” “Tryptophan Metabolism,” and “Lysosome” pathways (from Hallmark and KEGG compendia). These cross-omics connections indicate coordinated lipid-associated enzymatic and cellular processes that span both the metabolite and gene layers (Johnson and Stolzing 2019, Chung 2021, Mutlu *et al.* 2021, Ali *et al.* 2023, Watanabe *et al.* 2023, Chen *et al.* 2024, Jin *et al.* n.d.) (Data S12, available as supplementary data at *Bioinformatics* online).

Such findings suggest that aging is accompanied by systemic reprogramming of lipid metabolism, and that perturbations in lipid pathways may propagate across omics layers. For instance, alterations in polyunsaturated fatty acids and bile acid metabolism may reflect altered membrane dynamics (Boonstra *et al.* 1982, Pols *et al.* 2011, Vanni *et al.* 2019), proteostatis (Chiang 2013, Acosta-Montaño *et al.* 2019), and lipid signaling (Chiang 2013, Papackova and Cahova 2015), all of which are known to be associated with hallmarks of aging, including mitochondrial dysfunction, nutrient sensing, and chronic inflammation (López-Otín *et al.* 2023).

Interestingly, the gene-level annotation highlighted, among others, enrichment of Retinol Metabolism in younger subjects (Table 12, available as supplementary data at *Bioinformatics* online, KEGG). This was not explicitly captured at the metabolite level, and reflected decreased abundance with age of a varied list of metabolites not directly connected to Retinol metabolism, including Androsterone 3-glucuronide, Bilirubin, Thyroxine (T4), fatty acids that function as substrates for enzymatic oxidation pathways (arachidonic acid and linoleic acid) (Tallima and El Ridi 2018, Wang *et al.* 2021a), beside Retinol itself. The associated genes driving this enrichment, however, included an interconnected system of enzymes, ADH, CYP, UGT, and fatty acid/retinol metabolizing proteins (see Table 12, available as supplementary data at *Bioinformatics* online), which enables the processing of hormones, vitamins, and lipid signaling molecules by a combination of oxidation, reduction, and conjugation (Molotkov *et al.* 2002, Stevison *et al.* 2015, Rouleau *et al.* 2017, Yang *et al.* 2021, Gudas 2022, Lu and George 2024). The observed decrease in abundance of these metabolites and alteration in enzyme function with age likely contributes to impaired metabolic flexibility and increased oxidative stress, processes closely linked to aging and its associated functional decline.

Taken together, these findings align with the well-established role of lipid metabolism in aging and demonstrate the ability of *hypeR-GEM* to uncover biologically validated mechanisms, highlighting its effectiveness in real-world settings and underscoring its value for elucidating mechanistic insights into complex phenotypes such as aging.

## 4 Discussion

Enrichment analysis in metabolomics presents challenges for interpretation and multi-omics integration, arising from limited availability of curated, metabolite-centered signature repositories and from the absence of well-defined associations between metabolites and gene-centered biological knowledge and functional annotations. To address these challenges, we have developed *hypeR-GEM*, an R package that leverages gene–reaction–metabolite relationships from GEMs to enable gene set enrichment analysis on metabolomics data.

### 4.1 Evaluation and interpretation

We evaluated *hypeR-GEM* across 11 phenotypes from 10 paired metabolomics–proteomics and metabolomics–transcriptomics datasets. At the single-molecule level, 9 out of 11 comparisons showed statistically significant overlap between *hypeR-GEM*–predicted enzyme-coding genes and DE genes/proteins from the paired omics layer. Furthermore, seven out of these nine also demonstrated significant pathway-level concordance (*P* < .05) in at least one gene set database (Hallmark, KEGG, REACTOME, GOBP, and GOMF).

We emphasize that this evaluation is based on a specific assumption: that genes/proteins associated with DE metabolites are themselves DE. This assumption reflects the interconnectedness of metabolic reactions and gene expression, yet it is important to recognize both its utility and its limitations. We view this as a sufficient but not necessary condition for *hypeR-GEM*’s utility—i.e. we have intentionally set a stringent validation criterion, yet *hypeR-GEM* still identified meaningful associations. The observed concordance in the majority of phenotypes is noteworthy precisely because transcriptomics and metabolomics provide complementary but often decoupled information about cellular metabolism. As evidenced by our paired datasets, many differentially abundant metabolites lack corresponding differential expression in their associated genes, and conversely, many DE genes do not associate with differential metabolite abundance. This decoupling reflects the multiple layers of regulation between transcription and metabolite abundance—including post-translational modification, protein turnover, feedback inhibition, allosteric regulation, metabolite degradation, compartmentalization, and tissue-level heterogeneity.

### 4.2 Implications and scope of the tool

Given this fundamental decoupling, *hypeR-GEM* should be understood as an exploratory tool for hypothesis generation rather than a definitive validator of gene-metabolite functional relationships. The identified genes represent candidate regulators, enzymes, or responders to metabolic perturbations that merit further investigation. The tool’s utility lies in its ability to prioritize metabolite changes for mechanistic investigation by highlighting genes whose differential expression may be relevant to observed metabolic alterations.

We acknowledge that mechanistically linking metabolite changes to transcriptomic signals without additional data remains challenging. More complete understanding of metabolic dysregulation typically requires integration with fluxomics data (Winter and Krömer 2013, Cortassa *et al.* 2015, Emwas *et al.* 2022), which, under steady-state conditions, provides the only direct evidence that a metabolic pathway is actively engaged. Researchers seeking to establish causal or functional relationships should combine *hypeR-GEM* predictions with (i) domain-specific biological knowledge, (ii) targeted experimental validation, (iii) integration with flux measurements, or (iv) additional orthogonal omics approaches (e.g. proteomics, phosphoproteomics, or metabolic tracing).

An important consideration is that the metabolite-to-gene mapping via GEMs inherently restricts the analysis to metabolism-associated genes. Accordingly, pathway enrichment results obtained from *hypeR-GEM* should be interpreted as reflecting the metabolic component of biological processes, rather than the full pathway activity. However, many broader biological pathways that are not primarily defined by metabolic reactions, such as inflammatory response and cellular senescence, still contain enzyme-coding genes represented in the Human-GEM. For example, inflammatory pathways involve enzymes such as PTGS2 and ALOX5 through arachidonic acid metabolism, and cellular senescence pathways involve enzymes such as IDH1/2 and GSR through mitochondrial and redox metabolism. Consequently, *hypeR-GEM* is not intended to replace transcriptomics-based enrichment analysis, but rather to complement it by providing mechanistic insights into the metabolic layer of biological systems.

### 4.3 Practical application

In light of these observations, *hypeR-GEM* should be considered a valuable instrument for discovery and hypothesis formulation. By leveraging the systematic knowledge encoded in GEMs, the tool transforms metabolomic datasets into actionable biological hypotheses. Researchers can use the mapped enzyme-coding genes as a starting point for experimental design, pathway annotation, or computational validation, rather than as definitive mechanistic conclusions.

To demonstrate utility, we applied *hypeR-GEM* to age-associated metabolic signatures from the NECS study. This analysis revealed consistent enrichment in lipid-related pathways, including Lipid Metabolism, Fatty Acid Metabolism, Retinol, Tryptophan, Lysosome, and Bile Acid Metabolism. These results are consistent with the well-established role of lipid metabolism in aging, which contributes to mitochondrial dysfunction, dysregulated nutrient sensing, and chronic inflammation (Chiang 2013, Johnson and Stolzing 2019, Chung 2021, Chen *et al.* 2024, Jin *et al.* n.d.). These findings validate *hypeR-GEM*’s utility in analyzing complex biological mechanisms and underscore its capacity to generate mechanistically grounded hypotheses.

### 4.4 Implementation and extension considerations

In this study, we used Human-GEM as a comprehensive reference model to support the development and validation of a general metabolite-to-gene mapping framework. In addition, *hypeR-GEM* is flexible and can be readily applied to alternative GEMs if proper formats are provided, including tissue-specific or condition-specific models, providing a natural extension of the current workflow for more context-specific analyses.

*HypeR-GEM* is an open-source R package released under the GNU General Public License, and it is available for Linux, macOS, and Windows on GitHub. Comprehensive documentation, tutorial vignettes, and workflow examples are hosted at https://github.com/montilab/hypeR-GEM, and a versioned archive of the software is available through Zenodo (DOI: 10.5281/zenodo.20,586,748). In summary, *hypeR-GEM* offers a user-friendly tool designed to leverage the advantages of gene set enrichment analysis, thereby facilitating the interpretation of complex results from metabolomics studies.

## 5 Data and code availability

*EMT data*. The unprocessed data are publicly available through ProteomeXchange (Proteomics: accession PXD031071) and the National Metabolomics Data Repository (Metabolomics: PR001174), as described in Paul *et al.* (2023).

*Serine starvation data*. The RNA-seq data are available in the GEO (accession GSE286464), and the corresponding study is described in Jankowski *et al.* (2025). Metabolomics data has been deposited at Metabolomics Workbench; the access link remains pending publication in the Jankowski *et al.* study.

*ROSMAP data*. Study data were provided by the Rush Alzheimer’s Disease Center, Rush University Medical Center, Chicago. Data collection was supported through funding by NIA grants U01AG46161 (TMT proteomics), the Illinois Department of Public Health (ROSMAP). Additional phenotypic data can be requested at www.radc.rush.edu. Metabolomics data is provided by the Alzheimer’s Disease Metabolomics Consortium (ADMC) and funded wholly or in part by the following grants and supplements thereto: NIA R01AG046171, RF1AG051550, RF1AG057452, R01AG059093, RF1AG058942, U01AG061359, U19AG063744, and FNIH: #DAOU16AMPA awarded to Dr. Kaddurah-Daouk at Duke University in partnership with a large number of academic institutions. As such, the investigators within the ADMC, not listed specifically in this publication’s authors’ list, provided data along with its pre-processing and prepared it for analysis, but did not participate in analysis or writing of this manuscript. A complete listing of ADMC investigators can be found at: https://sites.duke.edu/adnimetab/team/. The Metabolon datasets were generated at Metabolon and pre-processed by the ADMC.

*M005 data*. The M005 proteomics and metabolomics data is available from the ELITE portal (https://doi.org/10.7303/syn69931740).

*COVID data*. The unprocessed proteomics data are publicly available through ProteomeXchange (Proteomics: accession PXD030662). The COVID-19-associated metabolite and protein signatures reported by Bi *et al.* (2022) are available in the supplementary materials of that study.

*NECS data*. NECS data will be available from the ELITE portal.

All analyses in this study were conducted using R, and the scripts are available from the GitHub repository: montilab/huang_hypeR.GEM_analysis: v1.0.0 (https://doi.org/10.5281/zenodo.17108624).

Any additional information required to reanalyze the data reported in this work is available from the lead contact upon request.

## Supplementary Material

btag588_Supplementary_Data
